# Can Achilles tendon xanthoma be distinguished from Achilles tendinopathy using Dixon method MRI? A cross-sectional exploratory study

**DOI:** 10.1186/s12891-021-04494-0

**Published:** 2021-07-16

**Authors:** Thomas Michael Zahradnik, Mark Cresswell, Kip Squier, Charlotte Waugh, Liam Brunham, Hazel Screen, Alex Scott

**Affiliations:** 1 Centre for Hip Health and Mobility, University of British Columbia, BC Vancouver, Canada; 2 Ottawa Health Research Institute, University of Ottawa, Ottawa, ON Canada; 3 Department of Radiology, University of British Columbia, Vancouver, BC Canada; 4 Centre for Heart Lung Innovation, University of British Columbia, Vancouver, BC Canada; 5 Bioengineering Division, School of Engineering and Materials Science, Queen Mary University of London, London, UK; 6 Department of Physical Therapy, University of British Columbia, Vancouver, BC Canada

**Keywords:** Achilles, Cholesterol, MRI, Ultrasound, Collagen

## Abstract

**Background:**

Familial hypercholesterolemia is a genetic condition characterized by life-long elevations of plasma low-density lipoprotein cholesterol. In addition to life-threatening cardiovascular complications, intratendinous cholesterol deposits (xanthomas) can lead to pain and tendon thickening, particularly in the Achilles. Clinical detection of xanthomas currently relies upon visual assessment and palpation, or ultrasound-based measures of tendon thickening or echotexture. Misdiagnosis of xanthoma can delay the commencement of potentially life-saving lipid-lowering therapy. Our primary purpose was to determine whether analysis of separated fat and water magnetic resonance images may be able to differentiate between xanthomatic and nonxanthomatic Achilles tendons through quantification of intratendinous fat content. The main hypothesis was that Achilles tendon xanthomas will demonstrate greater lipid content than Achilles tendinopathy or healthy control tendons.

**Methods:**

Bilateral MRI scans of Achilles tendons from 30 participants (n = 10 Achilles tendon xanthoma, n = 10 Achilles overuse tendinopathy, n = 10 healthy controls) were analyzed for total lipid content using the Dixon method of fat and water signal separation. Secondary outcome measures included tendon water content, as well as ultrasound characterization of tendon tissue organization and thickness.

**Results:**

Fat content was greater in Achilles tendon xanthomas compared to the tendinopathy (p < 0.0001) and control groups (p < 0.0001). Water content was also greater in Achilles tendon xanthomas compared to the tendinopathy (p < 0.0001) and control groups (p = 0.0002). Ultrasound tissue characterization revealed worse tissue organization in Achilles tendon xanthoma tendons compared to Achilles tendinopathy (p < 0.05) but demonstrated largely overlapping distributions. Achilles tendon xanthoma tendons were, on average, significantly thicker than the tendons of the other two groups (p < 0.01 and p < 0.001, respectively).

**Conclusion:**

MRI-derived measures of Achilles tendon fat content may be able to distinguish xanthomas from control and tendinopathic tissue. Dixon method MRI warrants further evaluation in an adequately powered study to develop and test clinically relevant diagnostic thresholds.

**Supplementary Information:**

The online version contains supplementary material available at 10.1186/s12891-021-04494-0.

## Introduction

Familial Hypercholesterolemia (FH) is the most commonly inherited cardiovascular disease (CVD), and is among the most common congenital metabolic disorders with a prevalence of 1:200 – 1:300 worldwide [1, 2] It is estimated that greater than 90% of those with FH have not been diagnosed [3]. Without treatment, individuals with FH are at significantly elevated risk of heart attack and other adverse cardiovascular outcomes [4]. Clearly, early detection of this condition before the onset of clinically manifested cardiovascular disease is crucial.

In addition to atherosclerotic plaque deposition, other areas of cholesterol deposition occur in FH: subdermally on and around the eyelids, as well as intra-tendinously in hands, elbows, knees and feet. Intratendinous deposits are most common in the Achilles tendon (ATX, Achilles tendon xanthoma) [5–7]. ATX deposition triggers an inflammatory response within the Achilles tendon leading to symptoms very similar to Achilles tendinopathy (ATY) [8]. Indeed, bouts of Achilles tendon pain lasting longer than three days are common in those with FH (cumulative prevalence of 46%) [9]. One study examined definite FH participants at the time of their diagnosis, and revealed that 26% (35/133) had previously seen a physician due to symptoms of Achilles pain, however none of these initial consultations led to a diagnosis of FH [9].

The accuracy of diagnostic imaging (magnetic resonance imaging [MRI] and ultrasound [US]) was systematically reviewed in 2019 [10]. There were 15 studies with 699 FH patients and 868 non-FH participants. Among non-FH participants, only 26 had documented Achilles tendon pathology (e.g. tear or tendinopathy). The review concluded that the ability of imaging to distinguish tendon xanthoma from other tendon pathology has not been firmly established. Whilst MRI and US were found to have acceptable sensitivity and specificity in detecting tendon thickening associated with ATX, this conclusion was based on low quality evidence, and currently available diagnostic criteria employed widely varying thresholds for the upper limits of Achilles tendon thickness (from 5.8 to 10.0 mm) [10].

There have been a few early attempts to quantify intratendinous fat signal using MRI, but none have established an imaging sequence that can distinguish xanthoma from healthy tendon or non-lipid-related tendon injury (e.g. tendinopathy). For example, Koivunen-Niemela et al. [11] evaluated a 0.1 Tesla fat–water discrimination technique: this method could not detect any differences in the fat or water signals between FH tendons and non-FH (overuse) tendinopathy. An alternative approach is the Dixon method, which generates images using combinations of in-phase and out-of-phase cycling of fat and water spin echoes: this method can be used to create four separate images (in-phase, opposed-phase, fat only, and water only) via summation and/or subtraction of water and fat signal. Utilization of the fat only image allows for water suppression such that fat infiltration can be quantified without distortion from water signal [12]. The Dixon method has proven to be effective in abdominal fat quantification [13], and recent research has supported its utility for fat quantification in the lower extremity, particularly to quantify lipid infiltration of calf musculature in patients presenting with Achillodynia [14]. Griffith et al. [15] have recently established, using magnetic resonance spectroscopy, that the Dixon method is able to detect cholesterol in the Achilles tendons, and that the tendons from people with FH display the predicted increase in fat signal compared to healthy tendons.

Given the above, the purpose of this pilot study was to determine whether the Dixon method of MRI may be able to differentiate between Achilles tendon xanthoma and Achilles tendinopathy through quantification of the intratendinous fat signal. This was an exploratory, cross-sectional, single-blind study comparing the radiological features of bilateral Achilles tendons from three groups of individuals – those with known FH-related Achilles tendon xanthoma, those with activity-related Achilles tendinopathy but no history of cardiovascular disease, and an additional control group with no tendon pathology or cardiovascular history.

## Methods

### Study design

This was a cross-sectional study comparing MRI findings among three groups of 10 individuals: ATX (Achilles tendon xanthoma), ATY (Achilles overuse tendinopathy) and ATN (Achilles tendon normal, i.e. asymptomatic control). US findings were also compared across the same groups.

### Participants

The study was performed in accordance with the relevant guidelines. All participants had to be: aged 25–50, fluent in English or able to provide an interpreter, and participating in at least 30 min of moderate or vigorous physical activity ≥ 3 times per week. ATX participants had to be diagnosed with definite FH (Dutch Lipid Clinic Network Score [DLCNS] of 8 or greater with ATX confirmed by physical examination in the medical record, and no diagnosis of Haglund’s deformity or other injury to the calcaneal tendon insertion. ATY participants had to be diagnosed with activity-related tendinopathy by a physician or other health professional, with a history of localized tendon pain lasting at least 3 days. ATN participants had to have no history of Achilles pain. ATN and ATY participants were excluded if they had a history of high circulating low density lipoprotein cholesterol (LDL-C) and/or total cholesterol (TC) levels. Additional exclusion criteria were: ineligible for MRI; pregnancy; prior surgery involving the Achilles tendon and/or associated musculature; inflammatory, genetic, musculoskeletal or metabolic condition influencing the Achilles tendon.

### Recruitment

ATX participants were recruited from the British Columbia Familial Hypercholesterolemia registry. All potential participants who met initial eligibility criteria were contacted by email to organize a follow-up call to gauge interest and to review eligibility criteria. ATY and ATN participants were recruited through word of mouth among the community of researchers and participants affiliated with the tendinopathy research program at the University of British Columbia. Participants who most closely matched the average anthropometric values of the ATX group were enrolled in the ATY and ATN groups. Recruitment continued until our target (n = 5 men and n = 5 women) was achieved for each group.

### Scanning procedures

Participants underwent both MRI and US on the same day (St. Paul’s Hospital, Vancouver, Canada). Achilles tendons were scanned bilaterally. Participants were requested to refrain from exercise for 48 h prior to scanning, and this was confirmed on the day of data collection.

Participants lay supine in the MRI machine (1.5 Tesla, GE Signa Twin-Speed, USA) with the ankle at approximately 20 degrees in a quadrature transmit-receive knee/ankle coil. Transverse proton-density fast spin echo sequences utilized a 3-point Dixon method in a 12 cm field of view (IDEAL; TE 26; TR 2778; echo train length 7; frequency encodes 256; phase encodes 224; NEX 2). Scans were 3 mm thick with a 1 mm interslice gap for a total of 34 slices covering 13.5 cm with 0.7533 mm^3^ resolution. Four images were generated, consisting of in-phase, opposed-phase, lipid-only, and water-only images.

The US instrument used was a 10-MHz linear-array transducer (Smartprobe 10L5; Terason 2000, Teratech, USA) held in a motorized tracking system (UTC Technology, Stein, Netherlands) that was mounted on a silicon-padded track that allowed for automated (hands-free) linear tendon ultrasonography over a distance of 12 cm[16]. The transducer, when oriented perpendicular to the Achilles tendon long axis, collected a transverse image every 0.2 mm. Software (UTC software v 1.0, UTC Technologies) was used to synchronize the image collection and the position of the ultrasound probe, allowing the location of each transverse scan to be known as described in the image analysis section below. Scans were collected in duplicate on each tendon, and the best of the two duplicates (i.e. free of artefact) was used for analysis. If both scans appeared to be of equivalent quality, one was chosen at random.

### Questionnaire

Participants reported their height, weight, age, dominant leg, and details of relevant medical and family history and medical or rehabilitation treatment. Those who had Achilles tendon pain at the scheduled time of their scan filled out the VISA-A (Victorian institute of sport assessment – Achilles) form, using a version modified for primarily non-sporting populations [17]. The VISA-A is a patient-reported outcome measure which can be used to gauge the severity of Achilles tendinopathy symptoms. Questions pertain to morning stiffness, pain (when stretching tendon, using stairs, after activity, during walking) and functional limitation, with a score of 100 indicating no pain/impairment, and lower values indicating higher pain/impairment. The initial values (in mmol/L) of TC and LDL-C in the participants’ FH registry were recorded, as well as the most recent on-treatment values.

### MRI analysis

**Fig. 1:**
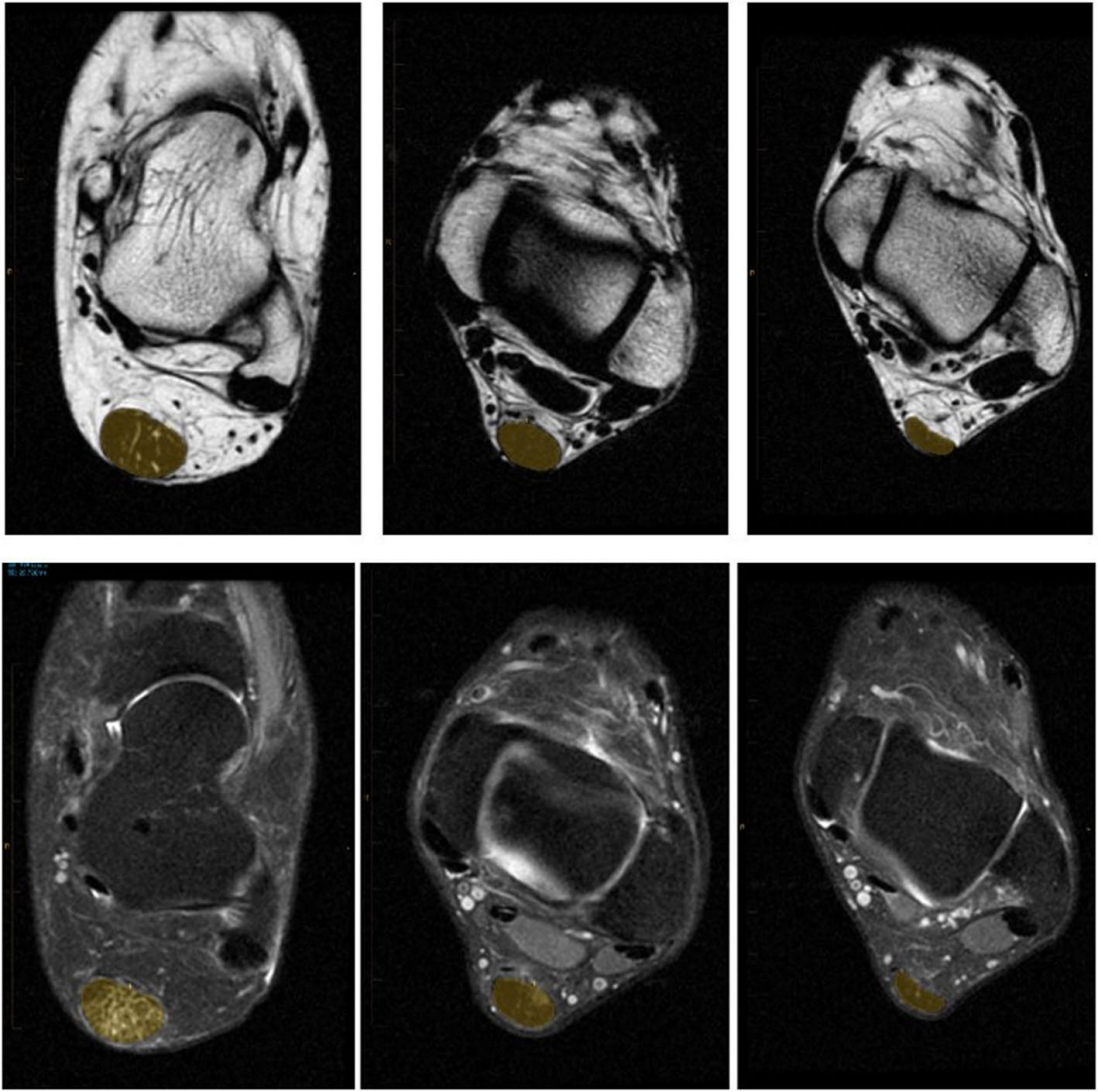
Lipid (top) and water-only (bottom) Dixon method transverse MRI of ATX (left), ATY (middle), and ATN (right) participants’ left ankle with Achilles tendon contouring shown in yellow. High signal (in white) indicates significant fat and water infiltration in the ATX patient image. A small area of water infiltration is seen in the ATY tendon, but with no corresponding fat signal. Anterior–posterior thickening is apparent in the ATX and ATY tendons

Each Dixon scan generated a DICOM file with 34 transverse slices each for four image series (in-phase, opposed-phase, lipid-only, and water-only). We used Inteleviewer (version 4–12-1-P451, Intelerad Medical Systems, Canada) to simultaneously view the four transverse image series, alongside a fifth sagittal-view window for orientation purposes. The calcaneal and gastrocnemius insertions were landmarked on the sagittal scan, and then the three transverse Achilles tendon scans which were closest to the midpoint of the Achilles tendon (i.e. midway between the two landmarks) were manually contoured, noting their distance from the calcaneus. This location of the tendon was chosen for analysis a priori as being the most common location for Achilles tendon pathology to occur. Next, lipid and water signal were range normalized for each tendon by contouring three consecutive slices of the Kager fat triangle and flexor hallicus longus muscle, and dividing the corresponding tendon fat and water signals by the resulting values. The normalization values were equivalent across the three groups for both fat (ATX 2689(497); ATY 2648(370); ATN 2713(375) and water (ATX 1268(261); ATY 1353(170); ATN 1336(188). The average Achilles tendon lipid and water signals in the contoured areas of the Achilles tendon (relative to the normalized signal) were then calculated, as per Griffith et al. [15] and multiplied by their cross-sectional area. Examples of contouring on the fat and water images are shown in Fig. 1. This yielded three data points, in arbitrary units, for each of Achilles tendon fat and water content (one from each evaluated transverse slice), which were averaged to yield a single value. Finally, the maximal anterior–posterior (A-P) thickness for each tendon was also recorded.

### Ultrasound tissue characterization

Each ultrasound scan generated a composite three-dimensional image file with 600 transverse slices over 12 cm; using ultrasound tissue characterization (UTC) software (v 1.0, UTC Technologies) the images could be navigated and viewed in sagittal, coronal and transverse views simultaneously. To locate the transverse sections of tendon which had been analyzed with MRI, the calcaneal insertion was identified and the three corresponding transverse scans of the Achilles tendon (spaced 4 mm apart, as on MRI) were manually contoured. The UTC software proprietary algorithm was then used to calculate the proportion of so-called Type I echoes within the contoured region of each of the three slices. The average of the three slices was used for statistical analysis.

### Statistical analysis

Statistical analysis was performed by a professional statistician using R (version 3.5.2, The R Foundation^©^). A linear mixed model was used for each of the following measures: lipid content, water content, echotexture, and antero-posterior thickness. Group (ATX, ATY or ATN) was the fixed effect, while individual variation was treated as a random effect. Data from each leg was included, and leg (dominant vs non-dominant) was also included as a paired (fixed) factor in the linear mixed model. A confidence level of 0.95 was utilized. *P* values of < 0.05, calculated via the Tukey method for multiple comparisons, were considered indicative of statistical significance. The data were inspected by a professional statistician and determined to be not in violation of the linear mixed models’ assumption of normality. Data are presented as means with standard deviation (SD). Among FH participants, the correlation between the average tendon lipid content with LDL-C and TC was examined using Pearson’s test with alpha of 0.05. The correlation between average tendon water content with LDL-C and TC was also examined using the same method.

## Results

### Participants

Demographics of the three groups are shown in Table 1. Recruitment was from June 2017 to September 2018. Of the 951 individuals in the registry patient population as of June 2017, 107 individuals were eligible based on a preliminary search filter (DCLNS ≥ 8, age, living in British Columbia, and active status in the registry). Of these, 38 individuals had a positive physical exam for ATX (55 others had a negative exam, and no exam was recorded for 14 others). Of the 38, the first 5 men and 5 women who expressed interest and who met all eligibility criteria were enrolled and comprised the ATX sample. The remainder of this paragraph describes the enrolled ATX sample. Mean DCLNS score of this was 16 (range 12–21). Corneal arcus and palmar/eruptive xanthoma were absent in all but two participants, while all participants had confirmed ATX. Xanthomas were present bilaterally in all participants. All ATX subjects had at least one high-cholesterol parent and seven participants had a family member (either a parent or grandparent) suffer a fatal heart attack with two participants having suffered a heart attack of their own (both males aged 31 and 37 at the time of onset). Six participants reported a treatment plan of healthy eating (low cholesterol diet) and exercise while all participants were prescribed at least one type of statin medication of varying dosage. In addition to statin, six participants were receiving biweekly injections of a PCSK9 inhibitor, either alirocumab or evolocumab. Mean baseline pre-treatment TC and LDL-C were 9.46 mmol/L and 7.56 mmol/L, which decreased to TC of 5.23 mmol/L and LDL-C of 3.60 mmol/L with lipid-lowering treatment.

**Table 1:** Participant demographics

	ATX (n = 10)	ATY (n = 10)	ATN (n = 10)
Age (year), mean ± SD	38 ± 7	40 ± 9	37 ± 7
BMI (kg/m^2^), mean ± SD	29 ± 4	26 ± 5	26 ± 4
Men	5	5	5
Women	5	5	5

*ATX* Achilles tendon xanthoma, *ATY* Achilles tendinopathy, *ATN* control, *BMI* Body mass index, No significant differences among groups (p > 0.05)

For ATY, word of mouth led to 23 individuals expressing interest in the study. Thirteen were ineligible due to age, different condition (calcaneal tendon insertion injury), and previous rupture or surgery. The remaining 5 men and 5 women expressed interest and were enrolled as the ATY sample, after confirming that they were demographically similar to the ATX group (i.e. the means of the groups’ age and body mass index (BMI) fell within 1 standard deviation of each other, and there were an equal number of men and women). All ATY participants were diagnosed by a registered physiotherapist, with onset of symptoms attributed to high impact exercise in 9 of the 10 participants (i.e. running, tennis) and long-distance walking in one participant. Duration of symptoms ranged from 0.5 to 11 years (mean, 2.6). All participants reported bilateral ATY symptoms. Rehabilitation approaches designed for participants involved stretching of the posterior chain musculature, strengthening of the posterior and anterior shank musculature and glutei, shockwave therapy, intramuscular dry needling, as well as “scraping” (an alternative medicine approach, gua sha). Recent blood testing revealed no history of hypercholesterolemia in seven participants while three (aged 25, 28 and 35) had never had their cholesterol levels assessed. No relevant medications were reported by ATY subjects, and no other medical conditions, genetic disorders or ailments existed amongst the group to their knowledge. No participant had a family history of FH. Six participants reported no family history of high cholesterol while three reported high cholesterol in one parent, and one participant reported high cholesterol in both parents.

Of 28 potentially interested ATN participants, we enrolled 5 men and 5 women that most closely resembled the demographics of the ATX group (i.e. the means of the groups’ age and BMI fell within 1 standard deviation of each other, and there were an equal number of men and women). No medical conditions or medications were reported by the control group. No participant reported any history of Achilles tendon pain or ATY symptomology. Eight participants reported normal blood cholesterol levels at the time of their most recent test while two, aged 26 and 32, had never been tested. Six subjects reported no family history of high cholesterol; however, four participants stated a history of high cholesterol in one parent.

### Achilles tendon symptoms

VISA-A scores of all participants who reported Achilles tendon pain at the scheduled appointment time are presented in the online data file. All ATY and four of the ATX participants reported varying degrees of bilateral achillodynia. Symptoms appeared, on average, less severe in the ATX group as indicated by the higher VISA-A score, but there were too few participants to enable a statistical analysis. The mean VISA-A score of the 4 ATX patients experiencing tendon symptoms was 87 (ranging from 76 to 93) compared to 66 (ranging from 44 to 83) in the ATY group.

### Tendon thickness

**Fig. 2:**
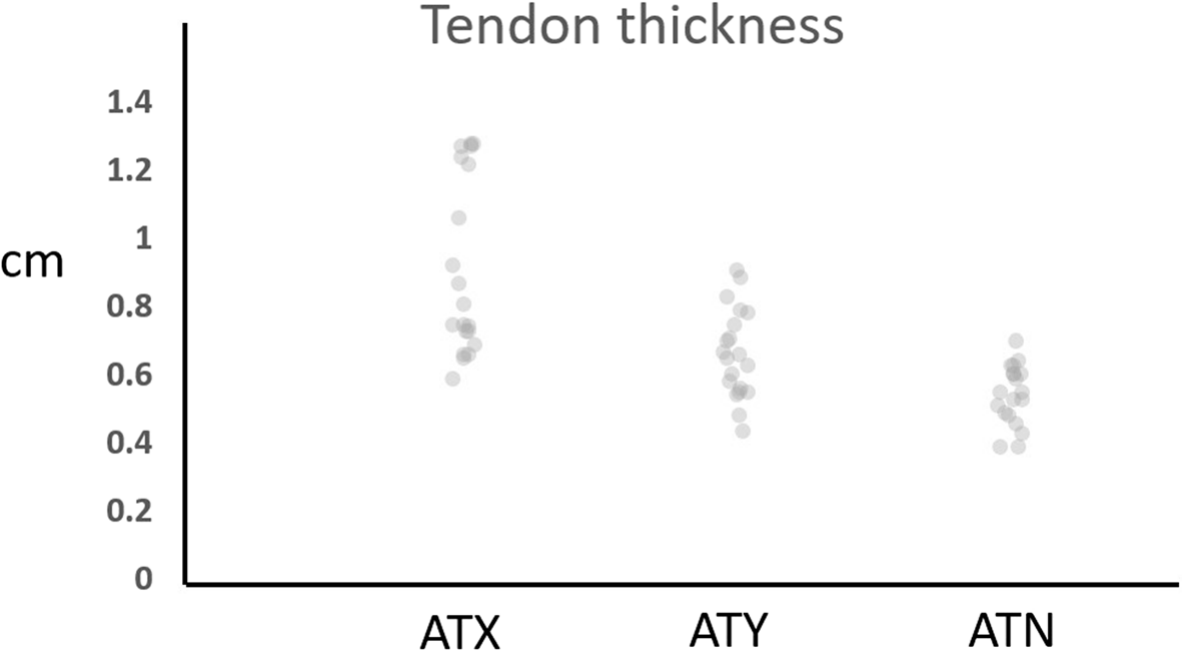
Tendon thickness (cm) was greater in those with xanthoma (ATX) compared to those with tendinopathy (ATY) or normal tendons (ATN), but the distributions demonstrated substantial overlap indicating that tendon thickness alone may be of limited diagnostic utility in separating normal from tendinopathic tendons

As measured on MRI, the antero-posterior thickness of ATX tendons, [9.1(2.6)mm] was significantly greater than the ATY [6.6(1.3)mm, p < 0.01] or ATN tendons [5.4(0.87)mm, p < 0.001] (Fig. 2). In transverse view, thickened tendons from both ATX and ATY groups took on an appearance of anterior convexity, as opposed to anterior concavity.

### MRI

**Fig. 3:**
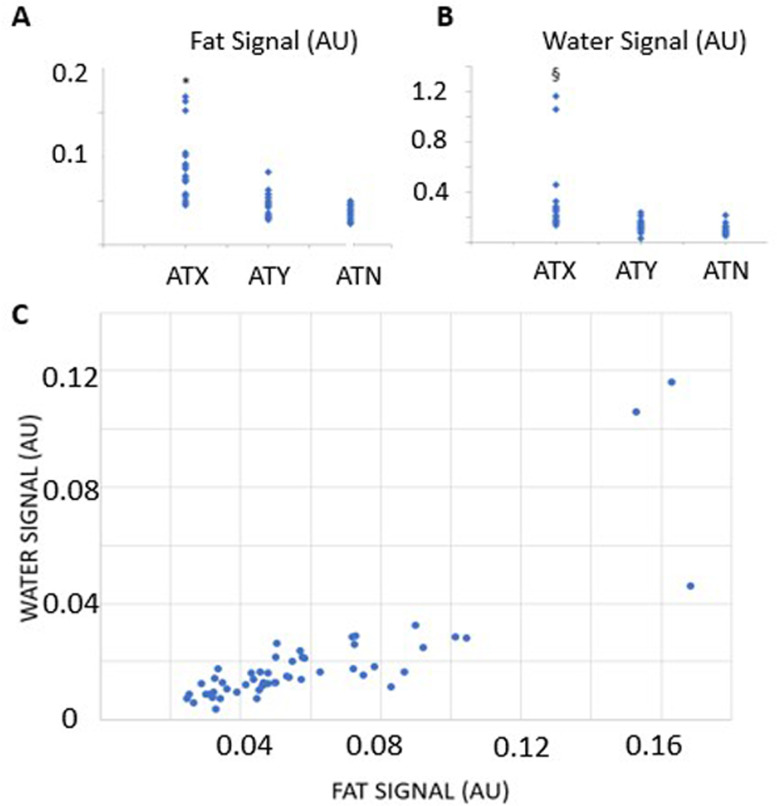
The MRI fat (**A**) and water (**B**) signal and their correlation (**C**). * indicates significantly different than ATY and ATN, p < 0.0001. § indicates significantly different than ATY (p < 0.0001) and ATN (p = 0.0002). The correlation between signals is strong (r = 0.827, p < 0.0001). AU – arbitrary units

The total lipid and water signals were higher in ATX tendons compared to ATY and ATN tendons (Fig. 3A and B)Total and relative lipid and water signals for the three groups are presented in Table 2. Total lipid (p = 1.2 × 10^−9^and water (p = 5.5 × 10^–6^) signals demonstrated significant group differences. The linear mixed model indicated a lack of group effect for relative lipid content (p = 0.20), whereas group was significant for relative water content (p = 0.042). There was a strong correlation between the fat and water signals, indicating that the accumulation of lipid was associated with worsening edema (Fig. 3C). Among the ATX group (n = 10), there was also a moderate correlation between total lipid content (average of right and left leg MRI) with LDL-C (R = 0.661, p < 0.05) and with TC (R = 0.6526, p < 0.05).

**Table 2:** Relative and total lipid and water values (in Arbitrary Units) of the ATX, ATY and ATN groups

	ATX	ATY	ATN
Relative lipid _(p=0.20)_	0.069 (0.025)	0.057 (0.014)	0.058 (0.0094)
Relative water _(p=0.042)_	0.24 (0.11)*	0.18 (0.041)	0.16 (0.029)
Total lipid _(p=1.2 x 10–9)_	0.086 (0.037)**	0.045 (0.014)	0.038 (0.0084)
Total water _(p=5.5 x 10–6)_	0.32 (0.28)***	0.13 (0.045)	0.11 (0.039)

^*^ ATX > ATN, p < 0.05 ** ATX > ATE or ATN, p < 0.001 *** ATX > ATE, p < 0.0005, ATX > ATN, p < 0.0001)

### Ultrasound tissue characterization

**Fig. 4:**
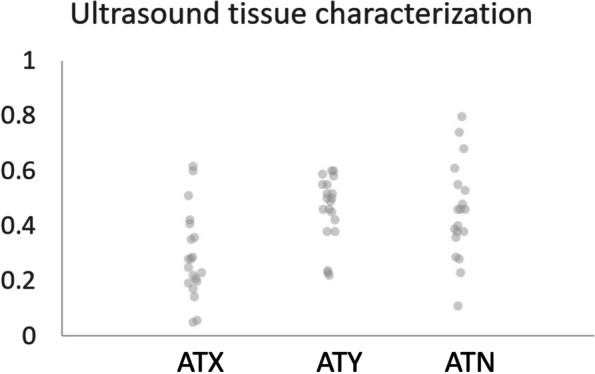
UTC analysis of ATX, ATY and ATN tendons. The wide range of values and overlap among groups may indicate a lack of diagnostic precision of this quantitative measurement technique

This analysis revealed a smaller percentage of Type I echoes in the ATX [29(16)%] than in ATY [46(12)%, p < 0.05] and a trend toward lower percentage in ATX than in the ATN tendons [45(17)%, p = 0.055], however the overlapping distribution of data points indicates that determining a diagnostic threshold is likely not possible (Fig. 4). An exploratory analysis revealed that a combined metric of (MRI fat signal / (Type I echoes) (i.e. accounting both for the amount of fat in the tendon and the level of collagen organization) was not better than fat signal alone at distinguishing ATX from ATY or ATN tendons.

### Missing data

The first control participant was only scanned unilaterally, leading to a total of 59 rather than 60 tendons included in the analyses.

## Discussion

Elevated fat content in Achilles tendon xanthomas (ATX) can be detected using Dixon method MRI. The fat signal was significantly higher in tendons from those with familial hypercholesteremia compared to those with overuse tendinopathy (ATY) or normal tendons (ATN). The generally non-overlapping distribution of fat values between hypercholesterolemic and normal tendons suggested that future work may be able to identify a clinically relevant diagnostic threshold. The mean + 2SD fat signal of ATN tendons was 0.0548, making 0.06 AU a potentially useful normal upper threshold which 8 of 10 ATX participants exceeded in at least one tendon. Only 2 of 20 ATY tendons exceeded this threshold. By comparison, the mean + 2SD tendon thickness of ATN tendons was 0.7158 cm, a value which was exceeded in at least one tendon by 10 of 10 ATX participants, and by 6 of 20 ATY tendons; suggesting the possibility that fat signal may be more effective than tendon thickness at discriminating ATX from ATY tendons. However, the overlapping distribution of fat signal in ATX and ATY tendons suggests that MRI alone may have limited diagnostic utility in separating xanthomatic from overuse Achilles pathology. As seen by the individual data points, despite the statistically significant difference there is substantial overlap in fat signal values between ATY and ATX tendons, indicating possible lipid deposition in association with some cases of overuse tendinopathy. Similarly, ultrasound tissue characterization was not able to distinguish ATX from ATY tendons, despite demonstrating significantly worse Achilles tendon collagen organization in people with FH.

ATX is an important diagnostic feature of FH, and the positive identification of ATX by physical exam alone can be challenging. The pathology can present as a spectrum including mild generalized thickening in the early stages. The current study identified the possibility of discriminating normal from hypercholesterolemic tendons through the use of MRI imaging, however the study population involved those with previously diagnosed, longstanding FH treated with a variety of lipid-lower agents which could have the potential to reduce tendon lipid content. A future adequately powered diagnostic or screening study with the Dixon sequences used in this study could be undertaken, being sure to enroll a larger group of individuals including newly diagnosed probable and definite FH. Such a study would allow greater understanding of the potential for MRI to improve the certainty of FH diagnosis. If the Dixon MRIs were found to have sufficient discriminatory power in this study, then it could become part of the standard diagnostic work-up for people with suspected FH, and for people with suspected tendinopathy who have concomitant dyslipidemia. However, such a study would be hampered by practical considerations such as the availability and expense of MRI.

Despite the small sample size of this pilot study, we were intrigued to find a strong correlation between tendon lipid content and both LDL-C and TC levels in the FH participants. This underscores the mechanistic link between circulating cholesterol, and lipid deposition within the Achilles tendon and suggests that the severity of xanthoma is related to the magnitude of dyslipidemia. A prospective study could be undertaken to ascertain whether reducing lipid levels therapeutically can lead to a reduction in tendon lipid deposits.

## Conclusion

This exploratory study has found that Dixon method MRI may be able to distinguish Achilles tendon xanthoma from overuse tendinopathy. Increasing fat accumulation was correlated with blood LDL-C and total cholesterol, and was associated with symptoms of tendon pain and functional limitation in a subset of patients.

## Supplementary Information

**Additional file 1.**

## Data Availability

The dataset supporting the conclusions of this article is included within the article (and its Additional file).

## Abbreviations

ATN: Achilles tendon normal
ATX: Achilles tendon xanthoma
ATY: Achilles tendinopathy
BMI: Body mass index
DLCNS: Dutch lipid clinic network score
FH: Familial hypercholesterolemia
LDL-C: Low density lipoprotein cholesterol
MRI: Magnetic resonance imaging
PCSK9: Proprotein convertase subtilisin/kexin type 9
TC: Total cholesterol
US: Ultrasound
UTC: Ultrasound tissue characterization
VISA-A: Victorian institute of sport assessment—Achilles
WHO: World health organization

## References

[1] Elis A, Zhou R, Stein EA (2011). Effect of lipid-lowering treatment on natural history of heterozygous familial hypercholesterolemia in past three decades. Am J Cardiol..

[2] Scott A, Zahradnik TM, Squier K, Beck C, Brunham LR (2019). Diagnostic accuracy of ultrasound and MRI for Achilles tendon xanthoma in people with familial hypercholesterolemia: A systematic review. J Clin Lipidol..

[3] Beeharry D, Coupe B, Benbow EW, Morgan J, Kwok S, Charlton-Menys V, et al. Familial hypercholesterolaemia commonly presents with Achilles tenosynovitis. Ann Rheum Dis. 2006;65(3):312–5. 10.1136/ard.2005.040766 PMC1798051 16176995

[4] Raju J, Norris J, Gaida J, Cook J, O’Neill S. Development and validation of the VISA-A(sedentary) questionnaire: a modified version of the VISA-A for nonathletic patients with Achilles tendinopathy. Online J Rural Nurs Health Care. 2017;17(1):S15.

[5] Outwater EK, Blasbalg R, Siegelman ES, Vala M. Detection of lipid in abdominal tissues with opposed-phase gradient-echo images at 1.5 T: techniques and diagnostic importance. Radiographics. 1998;18(6):1465–80. 10.1148/radiographics.18.6.9821195 9821195

[6] Fischer MA, Pfirrmann CW, Espinosa N, Raptis DA, Buck FM (2014). Dixon-based MRI for assessment of muscle-fat content in phantoms, healthy volunteers and patients with achillodynia: comparison to visual assessment of calf muscle quality. Eur Radiol..

[7] Marks D, Thorogood M, Neil HA, Humphries SE (2003). A review on the diagnosis, natural history, and treatment of familial hypercholesterolaemia. Atherosclerosis..

[8] van Schie HT, de Vos RJ, de Jonge S, Bakker EM, Heijboer MP, Verhaar JA (2010). Ultrasonographic tissue characterisation of human Achilles tendons: quantification of tendon structure through a novel non-invasive approach. Br J Sports Med..

[9] Youngblom E, Pariani M, Knowles JW. Familial Hypercholesterolemia. Adam MP, Ardinger HH, Pagon RA, Wallace SE, Bean LJH, Mirzaa G, et al., editors. Seattle (WA): University of Washington, Seattle. GeneReviews is a registered trademark of the University of Washington, Seattle. All rights reserved; 1993. (GeneReviews((R))).

[10] Tsouli SG, Kiortsis DN, Argyropoulou MI, Mikhailidis DP, Elisaf MS (2005). Pathogenesis, detection and treatment of Achilles tendon xanthomas. Eur J Clin Invest..

[11] Artieda M, Cenarro A, Junquera C, Lasierra P, Martinez-Lorenzo MJ, Pocovi M (2005). Tendon xanthomas in familial hypercholesterolemia are associated with a differential inflammatory response of macrophages to oxidized LDL. FEBS Lett..

[12] Nordestgaard BG, Chapman MJ, Humphries SE, Ginsberg HN, Masana L, Descamps OS (2013). Familial hypercholesterolaemia is underdiagnosed and undertreated in the general population: guidance for clinicians to prevent coronary heart disease: consensus statement of the European Atherosclerosis Society. Eur Heart J..

[13] Griffith JF, Hu M, Yeung DKW, Guo P, Lam SL, Xiao F (2017). Achilles Tendon Xanthomas: Fat-Water Separation at Baseline and after Treatment. Radiology..

[14] Goldberg AC, Hopkins PN, Toth PP, Ballantyne CM, Rader DJ, Robinson JG (2011). Familial hypercholesterolemia: screening, diagnosis and management of pediatric and adult patients: clinical guidance from the National Lipid Association Expert Panel on Familial Hypercholesterolemia. J Clin Lipidol..

[15] Bhatnagar D, Morgan J, Siddiq S, Mackness MI, Miller JP, Durrington PN. Outcome of case finding among relatives of patients with known heterozygous familial hypercholesterolaemia. BMJ. 2000;321(7275):1497–500. 10.1136/bmj.321.7275.1497 PMC27551 11118175

[16] Koivunen-Niemela T, Komu M, Viikari J, Alanen A. Magnetic resonance imaging of Achilles tendon xanthomas using a fat-water discrimination technique at 0.1 T. Acad Radiol. 1995;2(4):319–23. 10.1016/s1076-6332(05)80192-8 9419569

[17] Leyendecker JR, Brown JJ, Merkle EM. Practiceal guide to abdominal and pelvic MRI. Philadelphia: Lippincott Williams & Wilkins; 2010.

